# Diagnostic importance of serum markers in lung cancer

**DOI:** 10.3892/mmr.2026.13864

**Published:** 2026-03-30

**Authors:** Qichen Liang, Jianmei Song, Haixiang Wei, Lu Ning, Baoyu He, Ziteng Zhang

**Affiliations:** 1 School of Clinical Medicine, Jining Medical University, Jining, Shandong 272000, P.R. China; 2 Department of Operating Room, Affiliated Hospital of Jining Medical University, Jining Medical University, Jining, Shandong 272000, P.R. China; 3 Department of Thoracic Surgery, Affiliated Hospital of Jining Medical University, Jining Medical University, Jining, Shandong 272000, P.R. China; 4 Department of Laboratory Medicine, Affiliated Hospital of Jining Medical University, Jining Medical University, Jining, Shandong 272000, P.R. China; 5 Department of Thoracic Surgery, Qinghai Red Cross Hospital, Xining, Qinghai 810000, P.R. China

**Keywords:** serum peptide, serum markers, lung cancer, matrix-assisted laser desorption/ionization time-of-flight, early diagnosis, liquid biopsy

## Abstract

Lung cancer remains the leading cause of cancer-related mortality worldwide, which is largely because it is often diagnosed at an advanced stage. Early detection, particularly in high-risk populations, is important for improving patient outcomes. Liquid biopsy, which analyzes circulating biomarkers in the blood, offers a promising non-invasive approach for early diagnosis and monitoring. The present small-scale pilot study employed matrix-assisted laser desorption/ionization time-of-flight mass spectrometry-based serum peptidomics, which revealed preliminary differential peptide signals between patients with lung cancer and individuals with benign pulmonary nodules. These findings highlight the potential of this approach while underscoring the need for rigorous validation in larger cohorts. Integration of multi-analyte biomarker panels with clinical and imaging data represents a potential strategy for achieving more precise management of pulmonary nodules. Overall, serum-based biomarkers hold promise for advancing lung cancer diagnostics toward earlier detection and personalized patient management.

## Introduction

The International Agency for Research on Cancer estimates that one in five individuals will develop cancer during their lifetime, with one in nine men and one in 12 women succumbing to the disease (1). Lung cancer is the most commonly diagnosed cancer worldwide, accounting for 12.4% of cases, and remains the leading cause of cancer-related mortality, responsible for 18.7% of all cancer-associated deaths (1). The prevalence of smoking among men in densely populated countries such as China (41.5%), underscores the growing concern over escalating global lung cancer mortality rates (2).

Non-small cell lung carcinoma (NSCLC) constitutes ~85% of all lung cancer cases, with adenocarcinoma being the most prevalent histological subtype (3). Early-stage NSCLC is primarily treated with surgical resection, often supplemented with radiotherapy, chemotherapy or targeted therapies (4). However, metastatic recurrence occurs in 30–75% of patients despite these interventions (5,6). While patients at stage IA may achieve a 5-year recurrence-free survival rate of ≤82% (7), those with advanced disease experience a considerably lower 5-year overall survival rate of ~20%, largely due to metastases, even with the availability of targeted therapies and immunotherapy (8). Metastasis remains the primary barrier to long-term survival, as tumor cells often disseminate via the bloodstream or to target organs before clinical detection (9,10). Thus, early diagnosis and reliable postoperative evaluation are important for improving survival rates and reducing mortality. Low-dose computed tomography (LDCT) has enhanced early lung cancer detection but is associated with a high false-positive rate, with >10% of patients being misdiagnosed with cancer (11,12). Additionally, CT imaging struggles to differentiate post-radiotherapy pulmonary fibrosis from tumor recurrence and often fails to detect micrometastases, limiting its accuracy in assessing radiotherapy outcomes (13). Solid biopsies, although traditionally used for tumor evaluation, are invasive and unsuitable for real-time monitoring, emphasizing the need for less invasive diagnostic tools (14). Liquid biopsy offers a non-invasive, efficient and accurate alternative for tumor detection and monitoring. Analyzing the biological information present in blood enables real-time assessment and comprehensive profiling of cancer (14).

The present small-scale pilot study employed matrix-assisted laser desorption/ionization time-of-flight (MALDI-TOF) mass spectrometry (MS)-based serum peptidomics for detecting differential peptide signals between patients with lung cancer and individuals with benign pulmonary nodules. These findings highlight the potential of this approach while underscoring the need for rigorous validation in larger cohorts.

## Materials and methods

### Study rationale and design

To illustrate the practical application and inherent challenges of clinical peptidomics, a small-scale, preliminary study was performed. The primary aim was exploratory: To assess the feasibility of generating differential serum peptide profiles between malignant and benign pulmonary nodules. The study cohort consisted of 32 patients from the Affiliated Hospital of Jining Medical University (Jining, China) between January 2025 and June 2025; this included 29 patients with pathologically confirmed lung cancer (the cancer group) and 3 patients with benign pulmonary nodules (the control group). The inclusion criteria were patients aged 18–75 years with a confirmed diagnosis of lung cancer or benign pulmonary nodules. Exclusion criteria included patients with previous treatment (chemotherapy, radiotherapy or surgery), active infections, or any history of other malignancies. Benign nodule status was confirmed either by biopsy or radiographic stability over a period of ≥2 years. Key demographic and clinical characteristics of the participants are summarized in Table I. The present study was approved by the Institutional Review Board of the Institute of Medical Science, Affiliated Hospital of Jining Medical University (approval no. 2024-02-C011).

### Serum sample collection and preparation

Peripheral blood samples (5 ml) were collected from all participants. Serum was separated by centrifugation at 4°C for 10 min at 500 × g, then aliquoted and stored at −80°C until analysis. Upon receipt of peptidomic profiling, all samples were transported on dry ice and remained frozen. Visual inspection confirmed the absence of hemolysis or lipemia in all samples.

### Peptide extraction

Serum peptidome analysis, including peptide extraction, matrix-assisted laser desorption/ionization time-of-flight mass spectrometry (MALDI-TOF MS) analysis and quality control (QC), was performed by Hangzhou Well-healthcare Technologies Co., Ltd., using their proprietary platform. Peptides were extracted from serum using the Well-healthcare Serum Peptide Detection Kit according to the manufacturer's protocol. Briefly, 10 µl serum was mixed with 70 µl ultrapure water and added to a 96-well plate pre-loaded with nanoporous silica microspheres. The mixture was incubated at room temperature with agitation at 1,350 rpm for 15 min. After incubation, the supernatant was removed by centrifugation at 4°C for 10 min at 500 × g. The pellet was washed once with 80 µl ultrapure water and centrifuged again at 4°C for 10 min at 500 × g to remove the supernatant.

### MALDI-TOF MS analysis

The extracted peptides were eluted with a matrix solution containing acetonitrile and trifluoroacetic acid, mixed with an excess of α-cyano-4-hydroxycinnamic acid matrix. A 1-µl aliquot of the mixture was spotted onto a stainless-steel MALDI target plate and allowed to dry under vacuum. MS analysis was performed using a ClinMS-Plat I MALDI-TOF MS instrument (Hangzhou Well-healthcare Technologies Co., Ltd). Data were acquired in positive ion linear mode with the following parameters: Extraction voltage, 2.00 kV; detector voltage, 2.30 kV; delay time, 200 nsec. For each spectrum, 800 laser shots were accumulated across a mass range of 680-18,600 Da.

### QC

Rigorous QC measures were implemented. On each target plate, the following control spots were included alongside patient samples: One negative QC (N-QC; from pooled negative serum, prepared by mixing negative serum samples from healthy individuals), one positive QC (P-QC; from pooled positive serum, prepared by mixing serum from patients with lung cancer), and three baseline QCs (B-QC; from pooled negative serum, prepared similarly to N-QC). The positions of N-QC, P-QC, B-QC and calibration standards were randomly distributed on the target plate. QC samples underwent the same pre-treatment process as the test samples. System suitability required B-QC spectral similarity scores to be ≤0.34 (Euclidean and Manhattan distances), N-QC to test negative (score ≤0) and P-QC to test positive (score >0). All samples passed QC criteria, with a minimum of 500 laser shots and a minimum peak detection proportion of 50% per spectrum.

### Preprocessing and peak detection

Raw mass spectral data were preprocessed using MDAS PreData software (version 2.23; Well-healthcare Technologies Co., Ltd.). This included baseline correction, smoothing, peak alignment and peak picking. A total of 444 mass spectral peak signals were detected across all samples. These peaks were matched against the PeptideAtlas database (http://www.peptideatlas.org), resulting in the identification of 188 unique peptide sequences.

### Identification of differential peptide signals

For statistical comparison between the cancer (n=29) and control (n=3) groups, peptide peak intensity values were used. Peptide signals detected in <80% of the samples within either group were filtered out. Peptides with an absolute log2 fold-change (|log2FC|) >0.2 and P<0.05 were considered differentially expressed. No correction for multiple testing (for example, false discovery rate or Bonferroni) was applied due to the exploratory nature and small sample size of this pilot study.

### Functional annotation and enrichment analysis

The proteins corresponding to the identified differential peptides were subjected to functional annotation and enrichment analysis. Gene Ontology (GO) enrichment, Clusters of Orthologous Groups (COG/KOG) classification, and Kyoto Encyclopedia of Genes and Genomes (KEGG) pathway analyses were performed using InterProScan (https://www.ebi.ac.uk/interpro), KAAS (http://www.genome.jp/kegg/kaas/), UniProt (https://www.uniprot.org), and eggNOG (http://eggnogdb.embl.de). Protein-protein interaction (PPI) networks were constructed using the STRING database (https://string-db.org) and visualized with Cytoscape software (https://cytoscape.org).

### Statistical analysis

Categorical variables were presented as numbers and percentages. For comparisons between the cancer (n=29) and control (n=3) groups Fisher's exact test was used for all 2×2 contingency tables due to the small sample size, particularly in the control group. Differential peptides were identified using a non-parametric Mann-Whitney U test, suitable for comparing two independent groups with non-normally distributed data. P<0.05 was considered to indicate a statistically significant difference. All statistical analyses were performed using R software (version 4.1.3; Posit software), which can be accessed at https://www.r-project.org.

## Results

### Patient characteristics

The present preliminary feasibility study included serum samples from 32 patients. The cohort consisted of 29 patients with pathologically confirmed lung cancer (the cancer group) and 3 patients with benign pulmonary nodules (the control group), as detailed in Table I. There were no statistically significant differences between the two groups in terms of age distribution (P=0.851) or sex (P=0.548) (Table I). The majority of lung cancer cases were adenocarcinoma (24/29; 82.8%) and were at an early stage (T1a-T1c, 29/29; 100%). Detailed histopathological descriptions are provided in Table II.

### Serum peptidomic profiling and differential peptide identification

**Figure 1.**
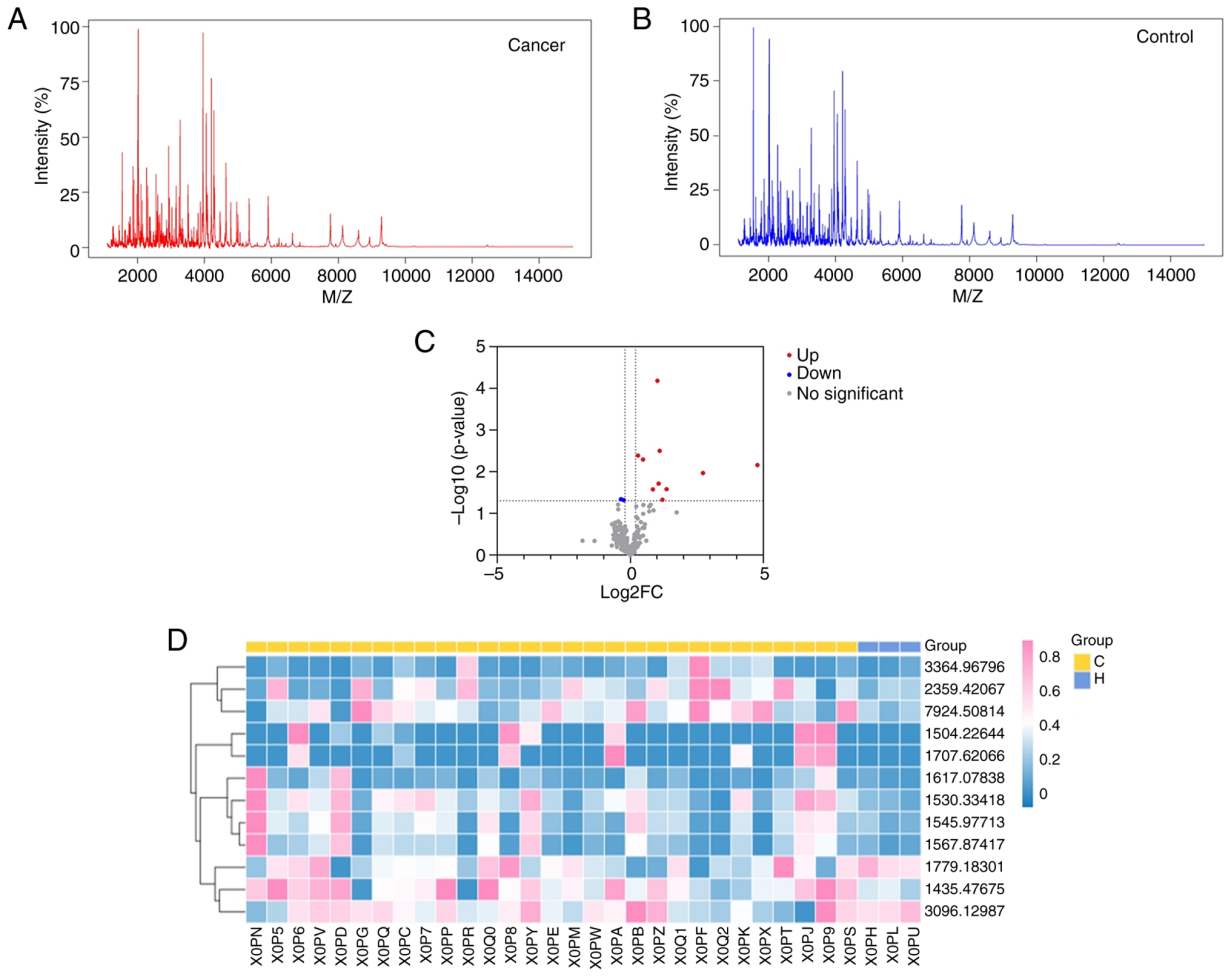
Preliminary analysis of polypeptide signals in pilot samples. (A) Mean spectra of the lung cancer-positive group. (B) Mean spectra of the lung cancer-negative group. (C) Volcano plot of differential polypeptide signals. The x-axis represents the log2FC, and the y-axis represents the -log10(P-value). Red dots indicate significantly upregulated proteins, blue dots indicate significantly downregulated proteins and gray dots indicate proteins with no significant change. (D) Differential polypeptide clustering heatmap. Red indicates higher expression, blue indicates lower expression and white indicates intermediate expression levels. Group C indicates lung cancer-positive samples; Group H indicates negative control samples. log2FC, log2 fold-change.

**Figure 2.**
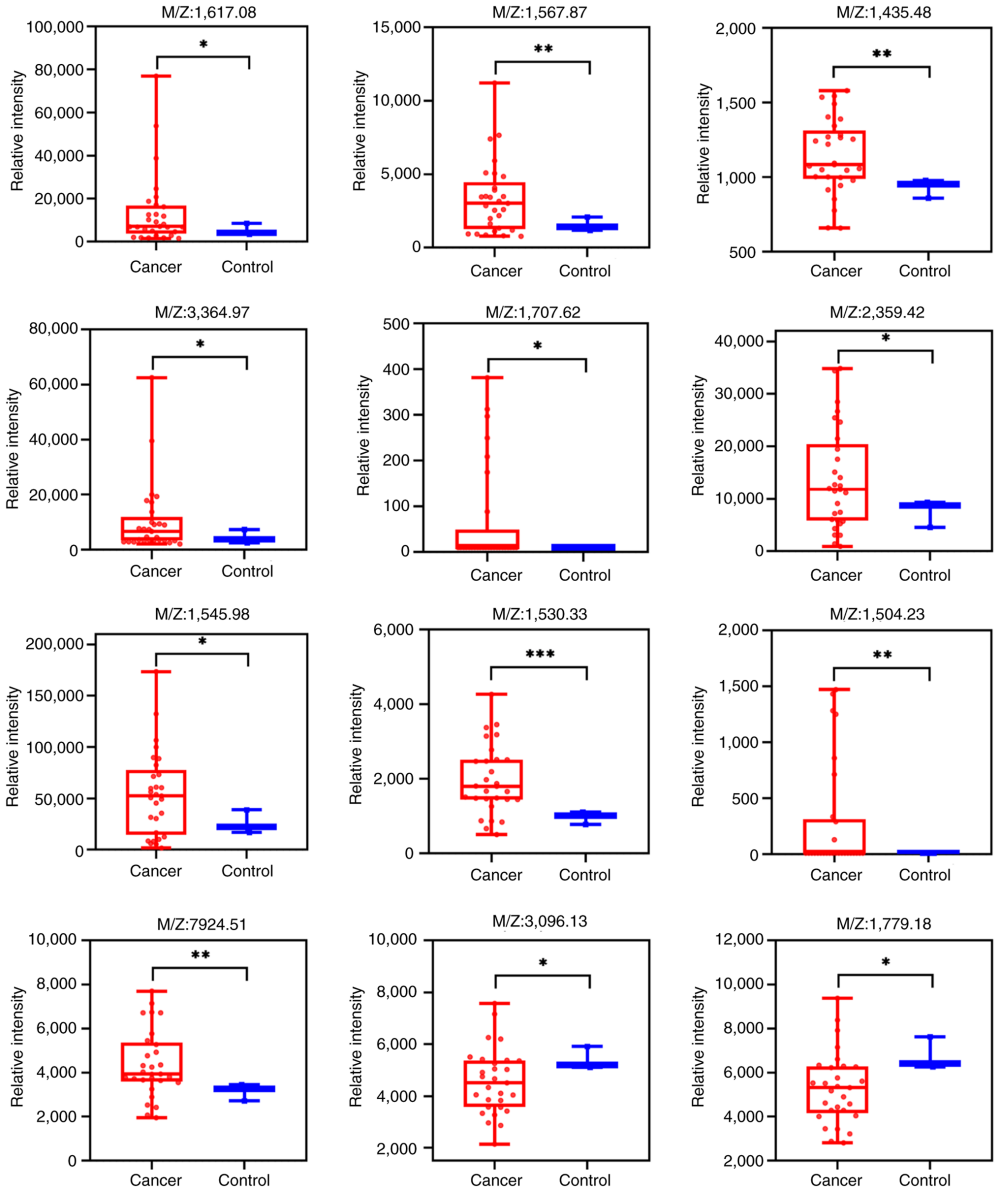
Differential polypeptide signal expression intensity box plots. *P<0.05, **P<0.01, ***P<0.001.

The average peptide spectra for the cancer (n=29) and control (n=3) groups are presented in Fig. 1A and B, respectively. Comparative analysis identified 12 peptide signals that met the pre-defined exploratory threshold for differential expression (|log2FC| >0.2 and P<0.05, Mann-Whitney U test; not corrected for multiple testing). As shown in the volcano plot (Fig. 1C), 10 peptides were upregulated and 2 were downregulated in the cancer group. The clustering heatmap (Fig. 1D) shows a general separation trend between the two groups, and the expression intensity distribution of these differential peptides is detailed in the box plots (Fig. 2).

### Biological annotation and pathway analysis of candidate proteins

**Figure 3.**
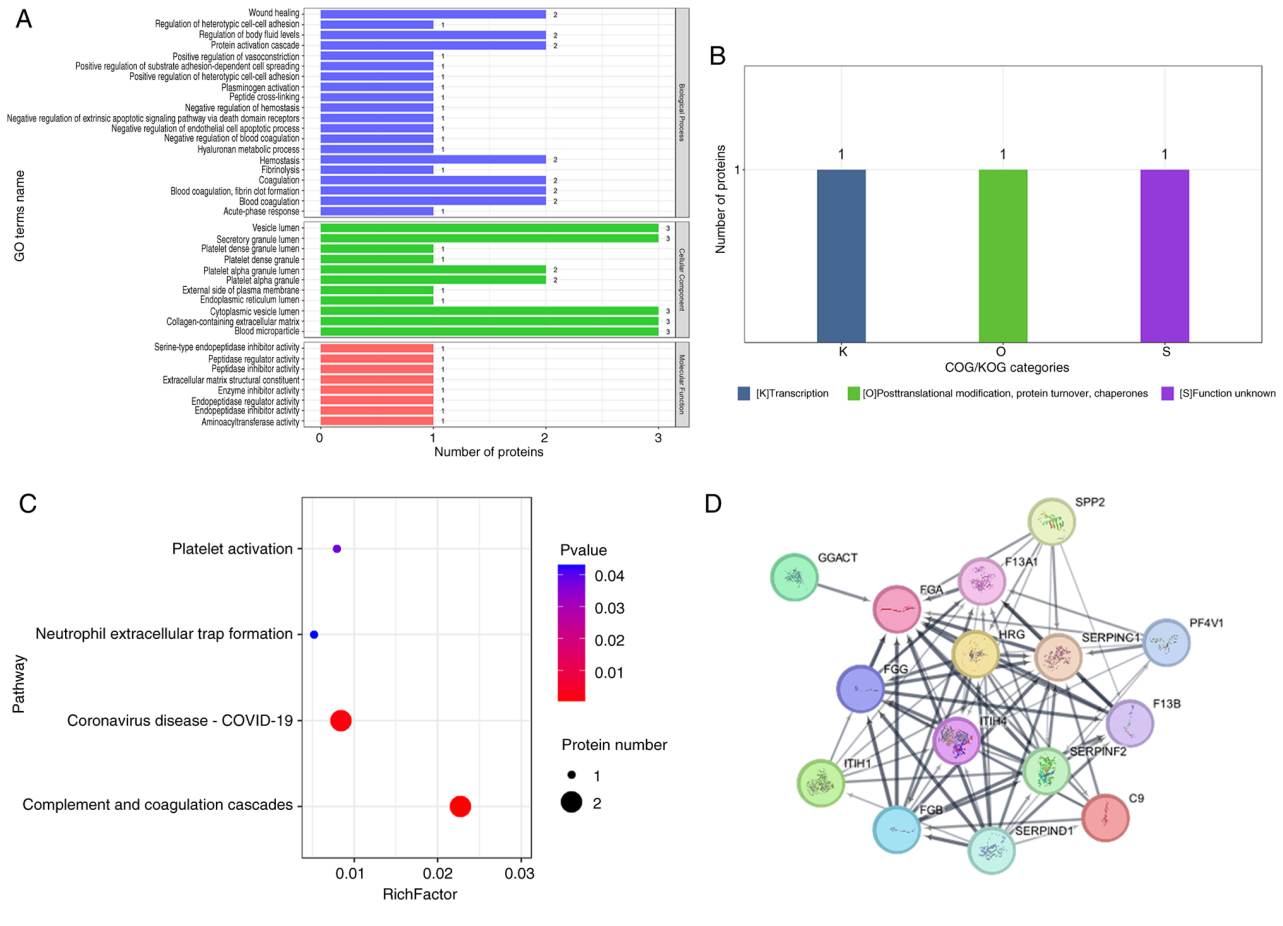
Biological analysis of proteins corresponding to differential polypeptides. (A) GO functional classification of differential proteins. The x-axis represents the number of differential proteins and the y-axis represents GO annotation entries. (B) COG/KOG functional classification of differential proteins. The x-axis indicates COG classification categories, and the y-axis represents the number of differential proteins. (C) Significantly enriched metabolic pathways for differential proteins. The y-axis displays the enriched pathways, and the x-axis represents the enrichment factor (RichFactor). The RichFactor is defined as the ratio of differential proteins to the total number of identified proteins annotated to the pathway; higher values indicate a larger proportion of differential proteins. Dot size corresponds to the number of differential proteins, and increasing red intensity indicates higher statistical significance. (D) Protein-protein interaction network. Each node represents a protein, and edges represent interactions, with line thickness reflecting interaction strength. GO, Gene Ontology; COG/KOG, Clusters of Orthologous Groups.

The 12 differential peptides mapped to three parent proteins: Fibrinogen α chain (FGA), inter-α-trypsin inhibitor heavy chain H4 (ITIH4) and coagulation factor XIII A chain (F13A1). GO enrichment analysis (Fig. 3A; Table SI) revealed that these proteins were primarily enriched in biological processes such as ‘platelet alpha granule lumen’ and ‘blood coagulation, fibrin clot formation’. COG/KOG functional classification (Fig. 3B) categorized the main function of these differential proteins under ‘Posttranslational modification, protein turnover, chaperones’ (Category O), Categories K (transcription), O (posttranslational modification, protein turnover, chaperones), and S (function unknown) were also observed. KEGG pathway analysis (Fig. 3C) indicated significant enrichment (P<0.05) in four pathways: ‘Platelet activation’, ‘Complement and coagulation cascades’, ‘Neutrophil extracellular trap formation’ and ‘Coronavirus disease-COVID-19’. PPI network analysis further demonstrated connectivity among these three candidate proteins (Fig. 3D).

### A conceptual framework for integrated diagnosis

**Figure 4.**
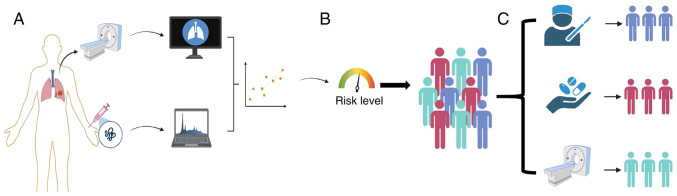
A conceptual framework for the future integration of serum biomarkers into lung nodule management. (A) Integration of clinical and radiological data with validated serum biomarker profiles. (B) Contribution of multi-modal information to a comprehensive risk assessment. (C) Facilitation of personalized management strategies. The depicted role of serum markers is contingent upon robust validation in future studies.

Based on the aforementioned findings, the present study proposes a conceptual framework for future pulmonary nodule management (Fig. 4). This framework envisages the integration of validated multi-analyte serum biomarker profiles with clinical and imaging data to achieve more precise risk stratification. The utility of this strategy is contingent upon rigorous future validation of the implicated biomarkers.

## Discussion

Th present preliminary exploratory study focused on the diagnostic potential of serum-based biomarkers in lung cancer, focusing on MALDI-TOF MS-based serum peptidomics.

In the exploratory peptidomic component of the present study, MALDI-TOF MS was applied, which preliminarily identified 12 peptides showing differential expression between lung cancer and benign nodules within a small sample set. These peptides map to three parent proteins: FGA, ITIH4 and F13A1. Functional enrichment analyses revealed notable involvement of these candidate proteins in pathways such as platelet activation, complement and coagulation cascades and neutrophil extracellular trap formation. These observations align with the well-established systemic hypercoagulable state and local inflammatory responses frequently observed in lung cancer (15,16). Tumor-associated thrombosis is a common complication in patients with lung cancer, potentially driven by coagulation-inflammation crosstalk that promotes disease progression and metastasis (17). FGA and F13A1, as core components of the coagulation cascade, may reflect tumor microenvironment remodeling and prothrombotic tendencies through their fragmented peptides (18,19); whereas ITIH4, an acute-phase protein, may represent a systemic host response elicited by the tumor (20). Thus, the preliminarily screened peptides in the present study provide initial clues and hypothesis-generating directions for exploring serum diagnostic biomarkers from the perspective of ‘coagulation-inflammation’ interplay in lung cancer.

However, it is crucial to emphasize the considerable limitations inherent in the present study, particularly its exploratory peptidomic component, which dictate the preliminary nature of its findings. The primary limitation is the extremely small sample size, particularly in the control group, which comprised only three individuals with benign nodules. This severe imbalance markedly reduces statistical power, rendering observed differences more likely attributable to inter-individual variation or chance rather than disease-specific signals. Secondly, owing to the constrained sample size, no correction for multiple testing was applied; the reported P-values carry a risk of false positives, and the results should be regarded as descriptive rather than confirmatory. Thirdly, the present study did not match or adjust for key clinical confounders such as age, smoking history or comorbidities, which can substantially influence the serum peptidome and confound disease-associated interpretations. Consequently, the principal value of this investigation lies in demonstrating the technical feasibility of the MALDI-TOF MS-based serum peptidomics workflow and generating a focused list of candidate peptides for prioritized evaluation in future large-scale validation studies, rather than proposing novel diagnostic biomarkers.

Looking ahead, translating serum biomarkers into routine clinical practice requires a concerted effort to address several key challenges beyond sample-size limitations. Priority must be given to adequate matching of relevant clinical confounders in case-controlled studies. More importantly, there is an imperative need for robust multicenter validation of single- and, more promisingly, multi-analyte panels in large-scale prospective cohorts. The future of cancer diagnostics lies in developing integrated diagnostic models that combine the genetic specificity of circulating tumor methylation, the rich proteomic information of the peptidome and exosomes, and the cellular insights from circulating tumor cells. Combining this multidimensional liquid biopsy data with established clinical and radiographic parameters will enable the development of reliable algorithms for personalized risk stratification.

The primary challenge in evaluating pulmonary nodules remains the need to facilitate timely intervention for malignancy while minimizing invasive procedures for benign disease. The high false-positive rate associated with LDCT underscores the need for more precise diagnostic approaches. The integration of imaging findings with emerging serum marker profiles, as conceptualized in this work, represents a promising pathway toward achieving superior risk stratification and personalized management. This integrated approach aligns with the principles of precision medicine, aiming to tailor interventions to each patient's unique and dynamic profile.

In conclusion, while serum biomarkers represent a frontier in lung cancer diagnostics with notable potential to improve early detection and patient outcomes, their journey from discovery to clinical utility is iterative and demands rigorous validation. By embracing a framework of methodological rigor, transparent reporting of limitations, as demonstrated in the preliminary study and collaborative validation in large trials, the field can move closer to making liquid biopsy an integral and reliable component of precision oncology for lung cancer.

## Supplementary Material

Supporting Data

## Figures and Tables

**Table I.** Clinicopathological characteristics of the patients recruited in the present study.

	Cancer (n=29)		Normal (n=3)		
			
Characteristic	N	%	N	%	P-value
Age, years					0.851
≥60	18	62.1	2	66.7	
<60	11	37.9	1	33.3	
Sex					0.548
Female	15	51.7	2	66.7	
Male	14	48.3	1	33.3	
Smoking history					0.095
Smoker	15	51.7	1	33.3	
Non-smoker	14	48.3	2	66.7	
Location					-
Right upper lobe	8	27.6	-	-	
Right middle lobe	4	13.8	-	-	
Right lower lobe	3	10.3	1	33.3	
Left upper lobe	10	34.5	1	33.3	
Left lower lobe	4	13.8	1	33.3	
Histopathology					-
Adenocarcinoma	24	82.8	-	-	
Squamous cell	2	6.9	-	-	
Small cell	1	3.4	-	-	
Other	2	3.4	-	-	
Tumor stage					-
T1a	9	31.0	2	66.7	
T1b	16	55.2	-	-	
T1c	4	13.8	-	-	
T2	-	-	1	33.3	

**Table II.** Patient pathology information.

Patient ID	Pathological information
D01	(Right) malignant tumor of the lung (upper lobe), inclined to carcinosarcoma, mass size 3×1.7×1.5 cm
D02	(Right) peripheral infiltrating adenocarcinoma (solid + alveolar) of the lung (upper lobe) with necrosis, mass size 2×1×1 cm
D03	(Right) peripheral infiltrating moderately-lowly differentiated adenocarcinoma of the lung (middle lobe; alveolar + micropapillary + solid), mass size 3×3×2 cm
D04	(Left) invasive adenocarcinoma (solid type predominant) of the lung (upper lobe), poorly differentiated, focal mucinous cell carcinoma, mass size 1.5×1.5×1 cm
D05	(Right) peripheral infiltrating moderately differentiated adenocarcinoma of the lung (upper lobe; predominantly alveolar, locally solid), mass size 1.8×1.8×1.2 cm
D06	(Upper lobe of right lung) peripheral type moderately-lowly differentiated squamous cell carcinoma, size 2×1.5×1.2 cm
D07	(Left) peripheral type invasive adenocarcinoma of the lung (lower lobe; alveolar + appressed + solid), mass size 2.5×1.5×1 cm
D08	(Left) peripheral type invasive adenocarcinoma of the lung (upper lobe; papillary + micropapillary + solid + adnexal), mass size 2×1×0.7 cm
D09	Microinvasive adenocarcinoma (upper lobe of the left lung), size 1×0.6×0.6 cm, no clear pleural invasion observed
D10	(Right) infiltrating highly differentiated squamous carcinoma of the lung (lower lobe), mass size 2×2×1 cm
D11	(Left) peripheral type invasive adenocarcinoma (alveolar + adnexal) of the lung (lower lobe), measuring 1×0.6×0.6 cm, without definite alveolar luminal dissemination and pleural invasion
D12	(Left) microinvasive adenocarcinoma of the lung (upper lobe), mass size 1×0.6×0.6 cm
D13	(Lower lobe of the right lung) lung tissue shows acute and chronic inflammatory cell infiltration, fibrous tissue proliferation and necrosis, and a fibrous connective tissue capsule wall with no obvious lining epithelium, accompanied by aggregation of surrounding tissue cells. The findings are consistent with a pulmonary hydatid cyst (echinococcosis) (Control group sample)
D14	(Left) peripheral infiltrating highly differentiated adenocarcinoma of the lung (upper lobe; alveolar + adnexal type), mass size 1×1×0.4 cm
D15	(Right) adenosquamous carcinoma of the lung (upper lobe; invasive moderately differentiated adenocarcinoma (alveolar type), ~50% of the tumor component + moderately - poorly differentiated squamous cell carcinoma, ~50% of the tumor component), mass size 1.2×1×0.5 cm
D16	(Left) widening of alveolar septa in the lungs (upper lobes) with interstitial fibrous tissue hyperplasia, lymphocytic infiltration and carbon dust deposition (Control group sample)
D17	(Left) Invasive adenocarcinoma (lower lobe of left lung; alveolar + papillary + adnexal), size 1.5×1×0.7 cm
D18	(Left) peripheral type invasive adenocarcinoma of the lung (upper lobe; alveolar + adnexal type), mass size 1.5×0.8×0.7 cm
D19	(Right) peripheral infiltrating adenocarcinoma of the lung (middle lobe; alveolar + adnexal type), mass size 0.7×0.6×0.5 cm
D20	(Right) invasive mucinous adenocarcinoma of the lung (lower lobe), mass 1 cm in diameter
D21	(Left) hypo-differentiated invasive adenocarcinoma of the lung (lower lobe; predominantly sieve-like structures and solid areas), size 1.7×1.5×0.8 cm
D22	(Right) peripheral type microinvasive adenocarcinoma of the lung (middle lobe) with a mass measuring 0.8×0.6×0.6 cm without definite pleural invasion
D23	(Upper lobe of left lung) neuroendocrine tumor, combined with immunohistochemistry and morphology consistent with small cell carcinoma (2 foci), the larger measuring 1.2×1×0.8 cm
D24	(Left) fine bronchial adenoma of the lung (lower lobe), size 0.8×0.8×0.4 cm; fibrous tissue hyperplasia and focal alveolar epithelial hyperplasia with carbon dust deposition were seen in the surrounding lung tissue (Control group sample)
D25	(Right) peripheral infiltrating moderately differentiated adenocarcinoma of the lung (lower lobe; predominantly alveolar, partially adnexal and papillary), mass size 1.5×1×0.6 cm
D26	(Right) microinvasive adenocarcinoma of the lung (upper lobe), mass size 0.7×0.6×0.5 cm
D27	(Right) microinvasive adenocarcinoma of the lung (upper lobe), mass size 1×0.8×0.6 cm
D28	(Left) highly differentiated invasive adenocarcinoma of the lung (upper lobe; a lepidic-predominant pattern and focal acinar components), mass size 1.5×0.7×0.7 cm
D29	(Left) peripheral type invasive adenocarcinoma of the lung (upper lobe; alveolar + adnexal type), mass size 2.5×1.3×1.3 cm
D30	Microinvasive adenocarcinoma (upper lobe of left lung), size 1.3×1.2×0.7 cm
D31	(Right) microinvasive adenocarcinoma of the lung (middle lobe), mass size 1.7×0.7×0.6 cm
D32	(Right) peripheral infiltrating moderately differentiated adenocarcinoma of the lung (upper lobe; alveolar predominant type), mass size 1.4×1×0.8 cm

## Data Availability

The data generated in the present study may be requested from the corresponding author. The data generated in the present study may be found in the ProteomeXchange Consortium under accession number PXD072901 at the following URL: https://proteomecentral.proteomexchange.org.

## Abbreviations

NSCLC: non-small cell lung carcinoma
LDCT: low-dose computed tomography
