# Supplementary material for: Diagnostic importance of serum markers in lung cancer
Source: Mol Med Rep. 2026 Mar 30;33(5):154. doi: 10.3892/mmr.2026.13864 (PMC13062724; doi:10.3892/mmr.2026.13864)
Supplement: Supporting Data [file Supplementary_Data.pdf]

Table SI. GO term enrichment analysis with associated P-values and protein counts.

Ontology	ID	Description	P-value	Gene ID	Count
BP	GO:0072378	blood coagulation, fibrin clot formation	2.02114200717404e-06	FGA/F13A1	2
BP	GO:0072376	protein activation cascade	2.87982191685195e-06	FGA/F13A1	2
BP	GO:0007596	blood coagulation	0.000417573883217471	FGA/F13A1	2
BP	GO:0050817	coagulation	0.000436388641147289	FGA/F13A1	2
BP	GO:0007599	hemostasis	0.000444029664318172	FGA/F13A1	2
BP	GO:0050878	regulation of body fluid levels	0.00110506659088314	FGA/F13A1	2
BP	GO:0042060	wound healing	0.00148164618870668	FGA/F13A1	2
BP	GO:0034116	positive regulation of heterotypic cell-cell adhesion	0.0022242220341202	FGA	1
BP	GO:0034114	regulation of heterotypic cell-cell adhesion	0.0038109311665554	FGA	1
BP	GO:0031639	plasminogen activation	0.00428661566808719	FGA	1
BP	GO:0042730	fibrinolysis	0.00428661566808719	FGA	1
BP	GO:0018149	peptide cross-linking	0.00444514351147707	F13A1	1
BP	GO:1902042	negative regulation of extrinsic apoptotic signaling pathway via death domain receptors	0.00492062608278832	FGA	1
BP	GO:0045907	positive regulation of vasoconstriction	0.00507908662324963	FGA	1
BP	GO:0030212	hyaluronan metabolic process	0.00539595723456754	ITIH4	1
BP	GO:2000352	negative regulation of endothelial cell apoptotic process	0.00571276055903258	FGA	1
BP	GO:1900026	positive regulation of substrate adhesion-dependent cell spreading	0.00666276688276157	FGA	1
BP	GO:0006953	acute-phase response	0.00761216788203289	ITIH4	1
BP	GO:0030195	negative regulation of blood coagulation	0.00761216788203289	FGA	1
BP	GO:1900047	negative regulation of hemostasis	0.00777034254446829	FGA	1
CC	GO:0072562	blood microparticle	3.71886222496803e-07	FGA/ITIH4/F13A1	3
CC	GO:0034774	secretory granule lumen	4.20662789648539e-06	FGA/ITIH4/F13A1	3
CC	GO:0060205	cytoplasmic vesicle lumen	4.32567721190062e-06	FGA/ITIH4/F13A1	3
CC	GO:0031983	vesicle lumen	4.36585378043221e-06	FGA/ITIH4/F13A1	3
CC	GO:0062023	collagen-containing extracellular matrix	9.97130846223969e-06	FGA/ITIH4/F13A1	3
CC	GO:0031093	platelet alpha granule lumen	3.34749139191826e-05	FGA/F13A1	2
CC	GO:0031091	platelet alpha granule	6.19489950293789e-05	FGA/F13A1	2
CC	GO:0031089	platelet dense granule lumen	0.00211065813339995	ITIH4	1
CC	GO:0042827	platelet dense granule	0.00316487256549591	ITIH4	1

CC	GO:0005788	endoplasmic reticulum lumen	0.0464821329953293	FGA	1
CC	GO:0009897	external side of plasma membrane	0.0572567921267388	FGA	1
MF	GO:0004867	serine-type endopeptidase inhibitor activity	0.016293503604569	ITIH4	1
MF	GO:0005201	extracellular matrix structural constituent	0.0266852460107492	FGA	1
MF	GO:0004866	endopeptidase inhibitor activity	0.0270038259294207	ITIH4	1
MF	GO:0030414	peptidase inhibitor activity	0.0281183081869292	ITIH4	1
MF	GO:0061135	endopeptidase regulator activity	0.029549963024077	ITIH4	1
MF	GO:0061134	peptidase regulator activity	0.0365289285716281	ITIH4	1
MF	GO:0004857	enzyme inhibitor activity	0.0613138734727035	ITIH4	1
MF	GO:0016755	aminoacyltransferase activity	0.0780085606054767	F13A1	1

MF, molecular function; CC, cellular component; BP, biological process; GO, gene ontology.
